# Estimation of Serum Calponin-2 in Patients With Breast Carcinoma and Its Clinicopathological Correlation

**DOI:** 10.7759/cureus.106200

**Published:** 2026-03-31

**Authors:** Shahbaz Habib Faridi, Ankur Rawat, Bushra Siddiqui, Sufia Naseem, Yogesh Rawat, Kiran Kalyan

**Affiliations:** 1 General Surgery, Jawaharlal Nehru Medical College, Aligarh Muslim University, Aligarh, IND; 2 Pathology, Jawaharlal Nehru Medical College, Aligarh Muslim University, Aligarh, IND; 3 Biochemistry, Jawaharlal Nehru Medical College, Aligarh Muslim University, Aligarh, IND

**Keywords:** biomarker, breast carcinoma, calponin-2, serum marker, tumor burden

## Abstract

Background: Breast carcinoma represents a major health burden among women in India. Identifying serum biomarkers like calponin-2 could aid in diagnosis, tumor burden assessment, and prognosis.

Objective: To estimate serum calponin-2 levels in patients with breast carcinoma and evaluate correlations with clinicopathological parameters.

Patients and methods: This prospective observational study was conducted at Jawaharlal Nehru Medical College, Aligarh Muslim University, from May 2023 to May 2025. Fifty histologically confirmed breast cancer patients were enrolled. Serum calponin-2 levels were measured pre- and postoperatively using ELISA. Clinicopathological features, including tumor size, grade, TNM stage, receptor status, and molecular subtype, were analyzed. Statistical analyses included paired *t*-tests and Pearson/chi-square tests.

Results: The mean patient age was 46.1 years, with the highest incidence in the 40-50 age group. Luminal B and HER2-enriched subtypes predominated. A significant postoperative decline in calponin-2 levels (mean reduction: 10.5%) was noted. Preoperative calponin-2 showed significant correlation with tumor size (ρ = 0.306, p = 0.030), T stage (ρ = 0.348, p = 0.015), and node positivity (ρ = 0.315, p = 0.021).

Conclusion: Serum calponin-2 correlates with tumor aggressiveness and shows promise as a tumor burden biomarker in breast carcinoma. It holds potential for integration into routine diagnostic panels, particularly in resource-limited settings.

## Introduction

Breast carcinoma represents a major global health challenge and continues to impose a substantial disease burden, particularly in low- and middle-income countries. Despite advances in screening and therapy, significant variability in disease presentation and outcomes persists, highlighting the need for improved diagnostic and prognostic tools.

Calponin-2 (h2-calponin) is an actin-binding protein involved in cytoskeletal organization, cellular motility, and proliferation. Increasing evidence suggests that calponin-2 may play an important role in breast cancer biology and has potential utility as a diagnostic and prognostic biomarker. Ji et al. demonstrated significantly elevated serum calponin-2 levels in patients with breast carcinoma compared to those with benign breast disease and healthy controls, particularly in hormone receptor-negative tumors and in patients older than 50 years, with a correlation to progesterone receptor status but not consistently with tumor size or nodal involvement [1].

Beyond its circulating role, calponin has been extensively studied as a myoepithelial marker in breast pathology. Comparative immunohistochemical studies have shown that calponin exhibits superior sensitivity compared to p63, smooth muscle actin (SMA), and CD10 in differentiating ductal carcinoma in situ from invasive carcinoma [2]. In rare breast tumors such as secretory breast carcinoma, calponin expression is typically absent, aiding in the distinction from benign mimics [3]. Similarly, invasive cribriform carcinoma lacks calponin expression, supporting its invasive classification despite its relatively favorable prognosis [4]. Solid papillary carcinoma, commonly occurring in elderly women, is usually hormone receptor-positive but calponin-negative, facilitating the differentiation from benign papillary lesions [5].

Conversely, benign breast entities such as collagenous spherulosis demonstrate strong calponin positivity around spherules, preventing misdiagnosis as malignancy [6]. In papillary breast lesions, continuous calponin staining supports benignity, whereas discontinuous or absent staining, often accompanied by loss of SMA, suggests atypical or malignant transformation [7]. These findings underscore the diagnostic relevance of calponin expression patterns in breast pathology.

While most existing studies have focused on tissue expression, interest in estimating the serum calponin-2 is emerging. In esophageal squamous cell carcinoma, elevated calponin-2 expression has been associated with a favorable prognosis and lower tumor grade [8]. Other cytoskeletal proteins, such as calpain-1, have also been linked to tumor grade, receptor status, and survival outcomes in breast cancer [9]. Debald et al. further identified calponin-2 as a potential serum marker for early detection of breast cancer, reporting elevated levels exclusively in malignant cases without any overlap with benign disease or healthy controls [10].

Breast carcinoma is the most common malignancy among women worldwide and a leading cause of cancer-related mortality [11]. In India, incidence has been steadily rising due to urbanization, delayed childbearing, reduced breastfeeding practices, and lifestyle transitions [12]. Importantly, Indian women tend to present at a younger age and with more advanced disease, often characterized by larger tumors and higher rates of lymph node involvement compared to Western populations [13]. Although established clinicopathological parameters such as tumor size, nodal status, histological grade, and TNM stage remain central to prognostication, outcome variability within similar stages has prompted the search for adjunct biomarkers that better reflect tumor biology [14,15].

This study aims to estimate preoperative and postoperative serum calponin-2 levels in patients with histologically confirmed breast carcinoma and to evaluate its potential role as a biomarker of tumor burden and aggressiveness. By correlating serum calponin-2 levels with clinicopathological parameters, including tumor size, histological grade, TNM staging, lymphovascular invasion, hormone receptor status (ER, PR), HER2 expression, Ki-67 index, and molecular subtype, this study seeks to assess the feasibility of serum calponin-2 as a reliable, non-invasive adjunct biomarker in breast cancer management, particularly within the Indian clinical context.

## Materials and methods

Study design and setting

This prospective observational study was conducted in the Department of Surgery, in collaboration with the Departments of Pathology and Biochemistry, Jawaharlal Nehru Medical College, Aligarh Muslim University, Aligarh. Patients with histopathologically confirmed breast carcinoma were enrolled and followed prospectively. Serum calponin-2 levels were measured both preoperatively and postoperatively without modifying standard treatment protocols.

Study duration and sample size

The study was carried out over 24 months (May 2023-May 2025). A total of 50 patients were included based on feasibility, resource availability, and comparability with similar biomarker studies [1,10].

Participants

Patients aged >14 years with confirmed breast carcinoma who provided written informed consent were included. Patients who refused consent, left against medical advice, or did not complete the required investigations were excluded.

Sampling and study groups

A non-probability consecutive sampling technique was used. As an observational study, patients served as their own controls, with within-subject comparisons of preoperative and postoperative serum calponin-2 levels.

Study parameters

Serum calponin-2 levels were correlated with clinicopathological parameters, including tumor size, nodal status, T and N stage, lymphovascular invasion, histological grade, and hormonal receptor status.

Sample collection and processing

Preoperative and postoperative venous blood samples (5 mL) were collected under aseptic conditions. Serum was separated by centrifugation and stored at −20°C to −80°C until analysis. Hemolyzed samples were excluded. Preoperative blood samples were collected prior to definitive surgical intervention. Postoperative samples were obtained two weeks following surgery, after resolution of the immediate postoperative inflammatory response, to minimize the effects of surgical stress on serum biomarker levels.

Serum calponin-2 estimation

Serum calponin-2 (CNN2) was estimated using a commercial sandwich ELISA kit (Human CNN2 ELISA Kit, ELK Biotechnology; Cat. No. ELK3394), following the manufacturer's instructions. The assay is based on antigen capture by immobilized antibodies, detection using a biotinylated antibody and streptavidin-HRP, and colorimetric measurement after TMB substrate addition. Optical density was read at 450 nm, and concentrations were calculated from a standard curve. All samples were analyzed in duplicate, and results were expressed in ng/mL (sensitivity: 0.114 ng/mL; detection range: 0.32-20 ng/mL).

Data collection and statistical analysis

Clinical, laboratory, and histopathological data were recorded using pre-designed proformas. Statistical analysis was performed using IBM SPSS Statistics for Windows, Version 24.0 (Released 2016; IBM Corp., Armonk, New York, United States). Continuous variables were expressed as mean ± standard deviation. Paired *t*-tests were used for pre- and postoperative comparisons. Correlation with clinicopathological parameters was assessed using Pearson’s correlation or chi-square test, as appropriate. A p-value <0.05 was considered statistically significant.

All classification systems and scoring methods used in this study, including TNM staging and histological grading, are internationally accepted standards and are free to use for academic and research purposes [14]. Hormone receptor status (estrogen and progesterone receptor), HER2 expression, and Ki-67 index were assessed by immunohistochemistry following established guidelines [15]. Serum calponin-2 estimation was performed using a commercially available Human CNN2 ELISA kit according to the manufacturer's instructions [10]. No proprietary tools or licensed scoring systems were used in this study.

Ethical considerations

Ethical approval was obtained from the Institutional Ethics Committee, Jawaharlal Nehru Medical College, Aligarh Muslim University, Aligarh. Written informed consent was obtained from all participants. The study adhered to the Declaration of Helsinki, and confidentiality was maintained throughout.

## Results

A total of 50 patients with histologically confirmed breast carcinoma were included in the study. The distribution of tumor stages revealed that the majority of patients presented with locally advanced disease. T3 tumors were the most common, accounting for 50% of cases, followed by T2 tumors in 28% of patients. Early-stage disease was less frequent, with T1 tumors observed in 10% of cases, while T4 tumors constituted 12% of the study population. The predominance of T2 and T3 tumors indicates delayed presentation and highlights the burden of advanced-stage disease at diagnosis (Table 1) [14].

**Table 1:** T-stage distribution in patients with breast carcinoma. Values are expressed as frequency and percentage. T stage was assigned according to the AJCC TNM staging system. AJCC: American Joint Committee on Cancer; TNM: tumor, node, metastasis.

T stage	Frequency	Percent
T1	5	10.0
T2	14	28.0
T3	25	50.0
T4	6	12.0
Total	50	100.0

The assessment of lymph node status showed that a substantial proportion of patients had nodal involvement. Overall, 68% of patients were found to be node-positive, underscoring the aggressive nature of the disease at presentation and its implications for prognosis and treatment planning (Table 2).

**Table 2:** Lymph node status in patients with breast carcinoma. Values are expressed as frequency and percentage. Lymph node status was determined on histopathological examination.

Lymph node status	Frequency	Percent
Negative	16	32.0
Positive	34	68.0
Total	50	100.0

Analysis of serum calponin-2 levels demonstrated a significant change following surgical intervention. The mean preoperative serum calponin-2 level was 8.83 ng/mL, which decreased to 7.90 ng/mL in the postoperative period. This reduction was statistically significant (p < 0.001), indicating a marked decline in circulating calponin-2 levels following tumor removal and suggesting a potential association with tumor burden (Table 3) [10].

**Table 3:** Preoperative and postoperative serum calponin-2 levels. Preoperative and postoperative serum calponin-2 levels were compared using paired *t*-test. The t value represents the test statistic for the mean difference, and the p value indicates statistical significance. Serum calponin-2 was measured using a commercially available ELISA kit. ELISA: enzyme-linked immunosorbent assay.

	Mean	Standard deviation	t value	p value
Preoperative calponin-2 (ng/mL)	8.833	1.54532	11.86	<0.001
Postoperative calponin-2 (ng/mL)	7.903	1.45844		
Δ Value (preop – postop)	0.930	0.555		
% Change	-10.52757	5.845490		

Further evaluation of the relationship between preoperative serum calponin-2 levels and clinicopathological parameters revealed significant correlations with markers of disease severity. Preoperative calponin-2 levels showed a positive correlation with pathological T stage (ρ = 0.348, p = 0.015) and tumor size (ρ = 0.306, p = 0.030). Additionally, a significant association was observed with lymph node positivity (ρ = 0.315, p = 0.021). These findings support the relevance of serum calponin-2 levels in reflecting tumor aggressiveness and disease extent in breast carcinoma (Table 4).

**Table 4:** Correlation of preoperative serum calponin-2 levels with clinicopathological features. Correlation analysis was performed using Pearson's correlation test. ρ represents correlation coefficient.

Variable	Statistical test	Correlation coefficient (ρ)	p value
T stage	Pearson correlation	0.348	0.015
Node positivity	Pearson correlation	0.306	0.030
Tumor size (cm)	Pearson correlation	0.315	0.021

## Discussion

This study evaluated serum calponin-2 levels in patients with breast carcinoma and examined their association with clinicopathological parameters. Our findings add to the growing evidence supporting the role of calponin-2 as a potential circulating biomarker in breast cancer, particularly in relation to tumor burden and disease aggressiveness.

The mean age of patients in the present cohort was 46.1 years, with the majority of cases occurring in the 40- to 50-year age group. This age distribution is consistent with previously reported Indian studies, which have shown that breast cancer in Indian women tends to present at a younger age compared to Western populations [11-13]. This earlier onset highlights the need for increased awareness and earlier screening strategies tailored to the Indian population.

A key finding of this study was the statistically significant reduction in serum calponin-2 levels following surgical intervention, with a mean postoperative decline of approximately 10.5%, which was statistically significant (p < 0.001). This observation suggests that circulating calponin-2 levels may reflect tumor burden and decrease following tumor removal. Similar findings have been reported by Debald et al., who demonstrated elevated serum calponin-2 levels exclusively in breast cancer patients, with no overlap with benign breast disease or healthy controls, supporting its tumor specificity [10]. Ji et al. also reported significantly elevated plasma calponin-2 levels in patients with breast cancer, particularly in older and progesterone receptor-negative women [1]. Collectively, these findings indicate that calponin-2 may serve as a dynamic biomarker that responds to changes in tumor load.

In our study, preoperative serum calponin-2 levels showed significant positive correlations with tumor size, pathological T stage, and lymph node positivity. These associations suggest a relationship between higher calponin-2 levels and increased tumor aggressiveness. Tumor size and nodal involvement are well-established prognostic factors in breast cancer, and their association with elevated calponin-2 levels strengthens the clinical relevance of this biomarker [14,15]. While Ji et al. reported limited correlation between calponin-2 levels and clinicopathological parameters [1], the stronger associations observed in our study may be attributed to differences in population characteristics, disease stage at presentation, or sample composition. Notably, a large proportion of patients in our cohort presented with locally advanced and node-positive disease, which may have amplified these correlations.

From a biological standpoint, calponins are actin-binding proteins involved in cytoskeletal organization and cellular motility. Calponin-2, in particular, has been implicated in processes such as cell migration, invasion, and tumor progression [8,9]. Overexpression of calponin-2 has been reported in several malignancies and may contribute to tumor cell invasiveness and stromal interactions [8]. Elevated circulating levels of calponin-2 may therefore reflect increased tumor activity, cellular turnover, or invasion in advanced disease.

The predominance of T3 tumors and high nodal positivity observed in this study reflects delayed presentation, a pattern commonly reported in Indian breast cancer cohorts [11-13]. The ability of serum calponin-2 to correlate with these adverse pathological features suggests its potential utility as a non-invasive adjunct marker for assessing tumor aggressiveness, especially in resource-limited settings where access to advanced molecular profiling may be restricted.

Although this study demonstrates promising results, certain limitations must be acknowledged. The sample size was relatively small, and follow-up was limited to the immediate postoperative period (two weeks), which restricts the ability to assess long-term outcomes such as recurrence, disease-free survival, and overall survival. Additionally, comparison with benign breast disease and healthy controls was not included. Therefore, the present findings should be interpreted as exploratory, and further studies with extended follow-up are required to establish the reliability of serum calponin-2 as a biomarker for long-term monitoring.

## Conclusions

Serum calponin-2 shows promise as a potential non-invasive biomarker in breast carcinoma. Its elevated preoperative levels and postoperative decline, along with correlation to tumor size, T stage, and nodal status, suggest relevance in gauging tumor aggressiveness and surgical response. Given the predominance of aggressive molecular subtypes in our Indian cohort, calponin-2 may enhance diagnostic and prognostic accuracy. Further large-scale studies are warranted to validate its clinical utility in personalized breast cancer management.
